# Model-based Recursive Partitioning for Survival of Iranian Female Breast Cancer Patients: Comparing with Parametric Survival Models

**Published:** 2017-01

**Authors:** Mozhgan SAFE, Javad FARADMAL, Jalal POOROLAJAL, Hossein MAHJUB

**Affiliations:** 1. Dept. of Biostatistics, School of Public Health, Hamadan University of Medical Sciences, Hamadan, Iran; 2. Modeling of Non-Communicable Diseases Research Center, Hamadan University of Medical Sciences, Hamadan, Iran; 3. Research Center for Health Sciences, Hamadan University of Medical Sciences, Hamadan, Iran

**Keywords:** Breast cancer, Parametric survival model, Recursive partitioning

## Abstract

**Background:**

Precise diagnosis of disease risk factors via efficient statistical models is the primary step for reducing the heavy costs of breast cancer, as one of the most highly prevalent cancer throughout the world. Therefore, the aim of this study was to present a recently introduced statistical model in order to assess its proficiency for model fitting.

**Methods:**

The information of 1465 eligible Iranian women with breast cancer was used for this retrospective cohort study. The statistical performances of exponential, Weibull, Log-logistic and Lognormal, as the most proper parametric survival models, were evaluated and compared with ‘Model-based Recursive Partitioning’ in order to survey their capability of more relevant risk factor detection.

**Results:**

‘Model-based Recursive Partitioning’ recognized the largest number of significant affective risk factors, whereas, all four parametric models agreed and unable to detect the effectiveness of ‘Progesterone Receptor’ as an indicator; ‘Log-Normal-based Recursive Partitioning’ could provide the paramount fit.

**Conclusion:**

The superiority of ‘Model-based Recursive Partitioning’ was ascertained; not only by its excellent fitness but also by its susceptibility for classification of individuals to homogeneous severity levels and its impressive visual intuition potentiality.

## Introduction

Breast cancer, as the leading cause of women’s cancer death throughout the world (1); is the second most common cancer among Iranian women (2). The progressive incidence of the disease and consequently its heavy imposed psychological and medical costs have enforced the health systems to search for efficient solutions in order to reduce this destructive burden. Surely, accurate diagnosis of protective and risk factors is the primary step for health care systems to improve their clinical decision making in therapeutic and care strategies (3). Statistical survival models are scientific tools to assess the proficiency of applied treatments and to survey the effectiveness of diagnosed medical indicators. Although, various classical survival techniques have been introduced to model time to death of breast cancer patients (4–6), but the superiority of machine learning algorithms has been proved recently in many survival studies (7–10). The higher precision of these novel methods has made them proper candidates to be compared with their traditional counterparts. ‘Model-Based Recursive Partitioning’ (MoBRP) is a special hybrid tree algorithm (11). MoBRP susceptibility for classification of individuals to homogeneous severity levels should be mentioned as its prominent ability, in addition to its impressive visual intuition potentiality.

Practically, the semi-parametric Cox proportional hazard (CPH) model is the most widely used representative of regression models for survival data (12). There have been designed a lot of studies for identifying the risk factors which are threatening the survival of Iranian women with breast cancer. Two pairs of model comparisons conducted; CPH versus shared frailty CPH, and CPH versus time-dependent CPH (6, 13). Parametric survival modeling also forms some parts of organized investigations (14). Further to these long-established models, some newly introduced learning algorithms have been used recently (15–17). However, the only application of MoBRP, in the field of survival modeling, refers to German breast cancer data (11).

Actually, this investigation was designed to assess the MoBRP capacity for identifying more relevant risk factors other than those recognized by previously applied parametric survival models. The MoBRP performance was evaluated and compared with proper survival models under the assumptions of four statistical distributions as the most frequently used distributions for time to event analysis. MoBRP has not ever been compared to the parametric survival models. Briefly, the aim of this study was to present practically the MoBRP in order to model the survival time of Iranian women with breast cancer.

## Materials and Methods

### Patients

The information of 1465 eligible Iranian women with breast cancer was used for this retrospective cohort study. Patients had been followed for nearly 30 yr, by the ‘Comprehensive Cancer Control Center’ of Shahid Beheshti University of Medical Sciences, Tehran, Iran. Although this center is placed in Tehran, but is a comprehensive center and responsible for admission of every referring patient; therefore, patients from different parts of Iran are participated this study. Patients were diagnosed and classified by the ‘International Classification of Diseases for Oncology 3^rd^ edition sites C50.0–C50.9’ and survival time was considered as the follow up period from surgical operation to the death of breast cancer. The applied dataset for this survey was heavy censoring such that 86% of patients did not experience the death of breast cancer within the follow-up period. This investigation participated factors include some baseline and pathological prognostic characteristics as age, ‘Human Epidermal growth factor Receptor 2’ (HER2), ‘Progesterone Receptor Status’ (PR), ‘Estrogen Receptor Status’ (ER)

### Model-Based Recursive Partitioning

Actually, MoBRP is a hybrid tree that refines classical modeling by the use of modern learning techniques of partitioning. Simply, if an overall model in the root node could not provide an appropriate fit to the total population, then observations are partitioned in a manner that a proper specific model could be associated with each terminal node. In addition, to MoBRP interpretability and precise prediction, its capability to recognize nonlinear relationships, has made it illustrious for analyzing complex structures (18). The participated covariates in MoBRP algorithm could be considered from two classes; partitioning covariates used for splitting, and model covariates used for node modeling. These two classes may be partially or completely the same (18). Following is the systematic MoBRP processes:

- A global model consists of model covariates, is fitted to the total population.
- The stability of estimated model parameters is assessed along each of the partitioning covariates. Afterward, the partitioning covariate associated with the most significant instability is chosen for splitting the total population. The stability assessments are according to the completely estimated model parameters.
- At this step, the splitting point for the partitioning variable is determined in such way that an objective function is minimized; this function could be the error sum of squares or negative log-likelihood of the tree calculated through all terminal nodes.
- The two previous steps are repeated at each terminal node and the tree would be grown.

### Statistical Analysis

Parametric survival models and MoBRP were fitted and their statistical performances were checked under the assumptions of four most common survival distributions (19–21); as exponential, Weibull, Log-logistic, and Lognormal. The effects of probable risk factors were assayed through these pointed statistical methods and homogeneous groups of Iranian breast cancer patients were formed by the use of MoBRP algorithm.

Since MoBRPs could be considered as high interaction models nested in parametric models, ‘Likelihood Ratio Test’ (LRT) was used to compare MoBRPs with routine survival models for each of the named distributions. Additionally, ‘Akaike Information Criterion’ (AIC) was employed to verify the supremacy between different models of different distributions.

## Results

The mean (SE) and median of survival time of female breast cancer patients were 4.16 (0.10) and 3.07 yr and the five-yr survival was 84% (95%CI: 81%–87%), respectively. The youngest participant was twenty yr old and the median of patients’ age was 54 yr. Approximately, 90% of individuals were older than 39 yr and according to the descriptive analysis 75.8%, 72.5% and 19% were ER*^+^*, PR*^+^* and HER2*^+^*, respectively.

Table 1 presents the results of model fitting. The first part of this Table regards to parametric model estimations; the significant negative coefficients of age and HER2 certify their adverse effects confirmed by all four models. Comparing parametric survival models for different statistical distributions, verified the minimum ‘AIC’ was attributed to Log-logistic model; following Weibull, Lognormal and then the exponential models. LRT confirmed the supremacy of Weibull to exponential model (LRT *P*-value=0.01).

**Table 1:** Comparison of models for different survival times distributions

	**Distributions Exponential**	**Weibull**	**Log-Logistic**	**Log-Normal**
Participated covariates in parametric models				
Intercept	10.04^**^ (0.33)	9.70^**^ (0.28)	9.46^**^ (0.29)	9.85^**^ (0.31)
Age	−0.02^*^ (0.01)	−0.01^**^ (0.01)	−0.01^**^ (0.01)	−0.02^**^ (0.01)
ER^+^	0.20 (0.26)	0.19 (0.22)	0.15 (0.22)	0.11 (0.24)
PR^+^	0.10 (0.26)	0.09 (0.21)	0.14 (0.22)	0.14 (0.23)
HER2^+^	−0.49^**^ (0.16)	−0.43^**^ (0.13)	−0.40^**^ (0.14)	−0.34^*^ (0.16)
Fitness Criteria of parametric models				
Model LogLikelihood	−2043.60	−2037.38	−2036.10	−2039.63
Model AIC	4097.17	4086.76	4084.20	4091.26
Fitness Criteria of MoBRP models				
Model LogLikelihood	−2029.88	−2033.58	−2031.22	−2023.09
Model AIC	4089.75	4085.15	4080.44	4074.19
Comparison of Models				
LRT p-value	< 0.01	0.05	0.02	< 0.01

* Significant at 5% level;

** Significant at 1% level;

PR^+^: being progesterone receptor positive breast cancer patient; ER^+^: being estrogen receptor positive breast cancer patient; HER2^+^: being epidermal growth factor receptor-2 positive breast cancer patient; AIC: Akaike Information Criterion; *MoBRP*: Model-Based Recursive Partitioning; LRT: Maximum Likelihood Ratio Test

**Fig. 1:**
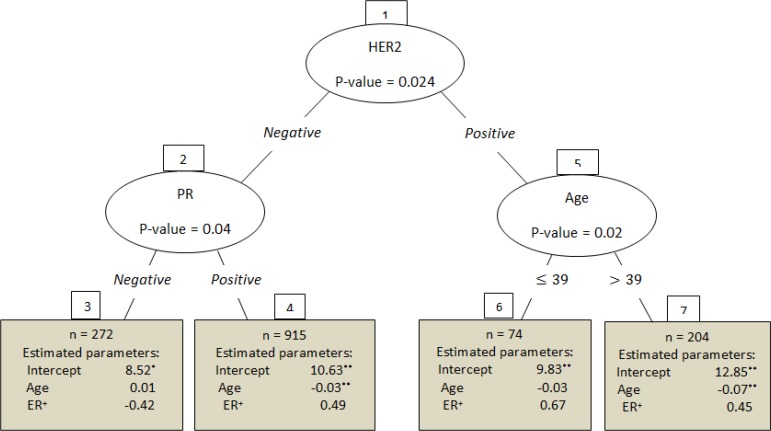
Model-based recursive partitioning, in the case of exponential survival time distribution *Significant at 5% level; **Significant at 1% level

In contrast to parametric models, LRT declared no significant differences between Weibull and exponential recursive partitioning. However, in accordance with parametric models, age and HER2 were known as effectual factors for survival modeling for each of the four distribution assumptions. Since exponential is the simplest survival distribution, Fig. 1 displays the tree partitioning in the case of exponential survival time assumption. As can be seen from this visualization; MoBRP was grown by splitting through HER2, PR and age; where ER and age were used for node modeling. Other than ER, all of the named covariates (as partitioning or modeling) were recognized significant affective risk factors for classification or modeling. Although, age was applied for classification and node model stability, but it was also significant within two of the four formed terminal nodes; such that demonstrated *P*-value*<* 0.01 for both of the second and fourth terminal nodes.

The second section of Table 1 that Lognormal, Log-logistic, Weibull, and exponential were, respectively the statistical distributions for which MoBRP provided lower ‘AIC’ and therefore, better fits for survival time of females with breast cancer.

The last section of Table 1 was dedicated to the comparisons of modern and classical models. Although the superiority of MoBRP was statistically proved by means of LRT for all four distributions, but this excellence had the most impression for Lognormal distribution; where the MoBRP had carried out its best fitness. The second most difference between MoBRP performance and parametric modeling was relevant to exponential distribution; the next places were allocated to Log-Logistic and Weibull, respectively.

Finally, the Lognormal recursive partitioning had the smallest AIC among the eight fitted models and, in this sense, was the best fitting model.

According to this tree-terminal-node fit, patients were classified by their PR and HER2 status; PR^−^ individuals formed the first terminal node while the remains, divided by their HER2 status, formed the second and third terminal nodes. The longest predicted survival time (i.e. 41.32 yr) was associated to the second terminal node, where the patients were PR^+^ and HER2^−^; versus, PR^−^ or PR^+^ and simultaneously HER2^+^ patients (i.e. the first and third terminal nodes) confirmed almost the same and lower length of survival time. Therefore, this best fitting model has introduced the simultaneously PR^+^ and HER2^−^ patients as the low risk group. In agreement with parametric models, this recursive partitioning also failed to recognize any significant effect for ER. This learning method additionally clarified that 22-yr acceleration in disease formation would cause a fraction of size 50% to the patients’ survival length time; In other words, the earlier creation of the breast tumor, for almost 22 yr, would reduce the behalf of survival time.

## Discussion

The better MoBRP fitness in comparison to parametric survival models is the most noticeable achievement of this study. In addition, to MoBRP possession of the same ability of parametric model fitting, it was capable to recognize hidden interactions and classified patients to homogeneous severity subsets. As can be seen from its prominent visual description, MoBRP identified one more prognostic factor (i.e. PR) addition to those recognized by proper parametric models. Since the effectiveness of this partitioning factor was previously certified in many clinical types of research regarding breast cancer prognostication in Iran (13, 22), therefore, the MoBRP more proficiency for detecting significant factors, was proved from the experimental perspective; the validation of this risk factor detection was also ascertained by LRT, from the statistical perspective. Following is a more detailed discussion of both medical and statistical aspects.

Regarding the resemblance of parametric model estimated parameters and their proximate AICs, all four models demonstrated the same performances as each other’s. The most observed difference between estimated parameters, associated to risk factors, was 0.15 and referred to HER2*^+^* under the two assumptions of exponential and Lognormal. Moreover, all models determined the protective or risk effect of factors, the same way. The effects of covariates, recognized as significant factors, have been confirmed by many previous researches. Surely, there are numerous studies proven the adverse effects of age and HER2*^+^* in the field of breast cancer (6, 13).

Additionally, the correct recognition of risk factors could be observed for all significant effects associated with terminal nodes of MoBRP. The estimated parameters were negative for every significant age effect (Fig. 1); introducing age as a risk factor.

Another worth noting marvel of MoBRP was its cut point selection of the age partitioning covariate (i.e. 39 yr). This cut point is almost the same as ‘40 yr’ chosen by many articles previously. The burden of disease bothers younger patients more than usual; In other words, they were divided the Iranian breast cancer patients to two subpopulations as young and old. The burden of the disease does not follow the common distribution, as Iranian patients with breast cancer are younger than the western countries (23). Therefore, “*Special programs should be considered for women under 40 yr old*” (2). This cut point was chosen according to experimental physicians’ experiences through some other researches (24, 25).

The split through PR, was the common feature of the current investigation and the previous exclusive applied of MoBRP algorithm, in the survival analysis (Fig. 1) (11). The information of 686 women from positive-node breast cancer was analyzed via the mentioned research. Patients were from German and eight prognostic factors were involved in MoBRP, two of them were used as model covariates and the remains, including age, ER, and PR, were employed as partitioning covariates. Weibull distribution was assumed for survival time and the tree was grown by just one split through PR. This two-terminal-node tree had nine parameters and its AIC was 1637.85. Unlike the current Iranian survey, in the German usage of MoBRP, the progesterone receptor was measured and treated as a numerical variable (i.e. fmol cytosol protein/mg) and the tree was subject to find the proper cut point for PR partitioning. Considering the MoBRP skill for finding cut points through maximizing likelihood, the available information on the current dataset only contains negative/positive state of patients’ PR; therefore, the binary partitioning would be certain after the selection of PR and this would limit the MoBRP excellent operation.

The most similar scientific method to current practical survey could be referenced to exponential tree (26). As is obvious by its name, the underlying exponential failure distribution was assumed for tree but the main difference between mentioned and current applied recursive partitioning is the statistical modeling in each node that is the exclusive ability of MoBRP. All the subjects in each node of the exponential tree have the same hazard rate, in other words, all the participated covariates in exponential tree are partitioning, while the hazard for subjects in a specific node of MoBRP could be different and would be determined by individual characteristics, which model the location parameter of the node distribution.

The selection of partitioning variable could be named as the second main difference between MoBRP and exponential tree. Although, MoBRP cut point selection is through maximizing likelihood but partitioning variable selection is according to the stability of estimated parameters. However, for exponential tree, variable and simultaneously optimal cut point selection is according to maximizing the likelihood of interval-censored survival times; simply, the exponential tree is grown by examining every allowable split on each covariate and therefore, numerous statistical tests and consequently selection bias are imposed to the algorithm (26, 27).

## Limitations

Unfortunately, the available patients’ medical records only contain their negative/positive status of PR, ER, and HER2, however, MoBRP was plausibly able to provide better fits if it was supported by the underlying numerical measurement of these risk factors.

## Conclusion

Our study reveals the newly introduced machine-learning algorithm, Model-based Recursive Partitioning, performed superior to the usual parametric models. Actually, the MoBRP potentiality to diagnose complex interactions and high order effects, supplemented with its impressive visual intuition has made it as a worthy complement in the context of survival model fitting. Moreover, its talent for regression modeling accompanied by simultaneous classification has famed it as an exclusive evolutionary fashion.

## Ethical considerations

Ethical issues (Including plagiarism, informed consent, misconduct, data fabrication and/or falsification, double publication and/or submission, redundancy, etc.) have been completely observed by the authors.
